# Food Resource Management and Healthy Eating Focus Associates with Diet Quality and Health Behaviors in Low-Income Adults

**DOI:** 10.3390/nu16132043

**Published:** 2024-06-27

**Authors:** Manije Darooghegi Mofrad, Briana M. Nosal, Daniela C. Avelino, Kate Killion, Michael Puglisi, Valerie B. Duffy, Ock K. Chun

**Affiliations:** 1 Department of Nutritional Sciences, University of Connecticut, Storrs, CT 06269, USA; manije.darooghegi_mofrad@uconn.edu (M.D.M.); briana.nosal@uconn.edu (B.M.N.); michael.puglisi@uconn.edu (M.P.); 2 Department of Allied Health Sciences, University of Connecticut, Storrs, CT 06269, USA; daniela_carolina.avelino@uconn.edu (D.C.A.); kate.killion@uconn.edu (K.K.)

**Keywords:** food resource management, healthy eating focus, diet quality, health-related behaviors, low-income, adults, COVID-19 pandemic

## Abstract

Nutrition education and food resource management (FRM) can assist food-insecure individuals in acquiring healthy and affordable food. We aimed to assess the relationships between FRM skills and healthy eating focus with diet quality and health-related behaviors in low-income adults during the COVID-19 pandemic. This cross-sectional study was conducted using an online survey of 276 low-income adults living in a low-food-access community in Northeast Connecticut. Through analysis of covariance, adults who usually or always had a meal plan, considered reading nutrition labels important, made a grocery list, were concerned about their food healthiness, and rated their diet quality as very good/excellent reported higher diet quality (frequency-based and liking-based scores) (*p* < 0.05 for all). Individuals who considered reading food labels very important and reported having a good diet reported less frequent pandemic-related unhealthy behaviors (consumption of candy and snack chips, soda or sugary drinks, weight gain, smoking) (*p* < 0.001). Furthermore, higher-frequency-based diet quality was associated with lower risk of overweight or obesity (OR: 0.37; 95% CI: 0.18, 0.76; *p*-trend < 0.01). Thus, FRM skills and healthy eating focus were associated with higher diet quality and healthier self-reported changes in diet, weight, and smoking behaviors during the pandemic.

## 1. Introduction

Low-income households have a higher risk of consuming poor diets, increasing their risk of various diet-related chronic diseases, notably obesity, cardiovascular disease, and diabetes [1]. Federal assistance programs like the Supplemental Nutrition Assistance Program (SNAP) have provided eligible low-income individuals and families with financial assistance to buy food [2], contributing to improving their food security [3,4]. However, on average, SNAP’s 42 million recipients have less healthy diets and are more obese than other Americans, including low-income Americans not on SNAP, indicating that extensive federal efforts to promote healthy diets need improvements [5,6]. Previous studies have demonstrated that appropriate nutrition knowledge is significantly related to healthy eating behaviors [7,8]. Furthermore, some studies indicated a connection between awareness and perception of dietary quality with realistic estimates of dietary intake [9,10]. Therefore, nutrition education programs can aid in improving diet quality by enhancing individuals’ knowledge of how to select healthy foods.

Food resource management (FRM) is a key component of nutrition education programs that promotes the ability to stretch food dollars and improves the ability to purchase affordable, nutrient-dense foods. FRM skills include using beneficial shopping practices such as using a grocery list, meal planning, and comparing food prices [11]. Nutrition education programs for low-income adults can improve FRM skills and, in turn, improve food security [12]. Therefore, it is important to understand the association of both nutrition knowledge and FRM skills as key components of nutrition education programs with diet quality in low-income individuals.

The COVID-19 outbreak challenged eating and lifestyle habits, potentially leading to less healthy behaviors during the pandemic [13]. Studies have shown that the COVID-19 pandemic had detrimental impacts on diet quality and food security in low- and middle-income countries [14]. The importance of FRM increased during the pandemic due to lower budgets and elevated food prices. In 2019, nearly 10% of American families faced financial challenges or limited resources to satisfy their food needs [15], and this percentage reached an alarming 38% in April 2020 [16]. Therefore, it is necessary to assess the association of FRM skills and nutrition awareness with changes in eating behaviors during the pandemic.

The present research involves a study of a low-income and low-food-access area in Northeast Connecticut. Specifically, Windham County was chosen since this area has the lowest median income [17] and the highest prevalence of food insecurity and poverty [18]. In our recent nutrition assessment conducted at the mobile food pantry distribution sites in Northeast Connecticut just before the COVID-19 pandemic, mobile food pantry users had significantly lower quality diets than the mean US population [19]. Most participants consumed low amounts of vegetables, fruits, whole grains, and dairy and high amounts of sugar and saturated fatty acids. A minimum of 30% of participants had vitamins A, E, C, folate, calcium, magnesium, and zinc intakes below the estimated average requirement (EAR). We also found that those with hypertension reported the highest consumption of dairy and added sugar, with a higher intake of added sugar from sugar-sweetened beverages [20]. Thus, understanding the impact of the COVID-19 pandemic on their eating habits and body weight status and how FRM and healthy eating focus may improve these pandemic-related behaviors will provide meaningful information for future nutrition interventions.

There is limited research on the associations between FRM skills, healthy eating focus, and diet quality among low-income, low-food-access populations. Therefore, the purpose of this study was to examine whether FRM skills and healthy eating focus were associated with diet quality scores, and whether these skills and knowledge were associated with self-reported changes in health-related behaviors during the pandemic among low-income adults in Northeast Connecticut. We also aimed to assess the association between diet quality and self-reported prevalence of obesity, diabetes, and hypertension.

## 2. Materials and Methods

### 2.1. Study Overview

This study was a cross-sectional online survey conducted between February and April 2022. The study included a convenience sample comprising 276 adults who live in low-income, low-food-access communities in Connecticut as designated by the USDA’s Food Access Research Atlas database [21]. The study design has been published in detail [22]. In brief, participants were invited to take part in the survey through advertisement flyers containing a QualtricsXM (https://www.qualtrics.com, accessed on 21 June 2024) survey link and a QR code, which were distributed in common areas, left in mailboxes, and given out to pantry users and community-based low-income food and resource agencies. Participants were also recruited via email from low-income housing communities, food pantries, and community organizations, as well as from a private Facebook group in Connecticut focused on the relevant zip codes. To participate in the survey, individuals had to be over 19 years old, reside in the three specific zip code areas, and be able to read and speak English and/or Spanish. Participants took the online survey on their own personal devices such as smartphones, tablets, or laptops, in either English or Spanish. The study protocol was approved by the University of Connecticut Institutional Review Board (X22-0013) and all participants provided written informed consent.

### 2.2. Assessment of Sociodemographic, Health, and Lifestyle Characteristics

These data included information about where the participants were born, how long they have lived in their current location, gender, primary language, race/ethnicity, marital status, educational level, household size, and employment status.

To assess weight status, participants reported their body shape using a 9-point Figure Rating Scale [23], where 1 to 2 were underweight, 3 to 4 were normal weight, 5 to 6 were overweight, and 7 to 9 were obese. Participants’ diabetes and hypertension status were self-reported, asking participants if they had been medically diagnosed by a physician. Smoking habits, cigarette addiction level [24], and physical activity [25] were evaluated with a self-reported questionnaire.

### 2.3. Diet Quality Assessment

The English/Spanish Short Healthy Eating Index (sHEI) Survey [26], a frequency-based measure, and the Liking-based Diet Quality Index (Liking-DQI) Survey were used to assess dietary quality [27,28,29]. The sHEI survey is a simple and reliable tool that evaluates diet quality by asking participants about the frequency of eating different food groups, such as vegetables, green and starchy vegetables, fruits, fruit juice, grains, whole grains, beans, nuts/seeds, seafood, milk, low-fat milk, sugar-sweetened beverages, added sugars, water, and saturated fats. It has been shown that the total sHEI score is highly correlated with the 24 h recall HEI score (r = 0.79) [26]. The frequency of food consumption was compared with the U.S. Dietary Guidelines for Americans [30] and weighted to estimate sHEI according to a published sHEI scoring system [26].

The Liking-DQI Survey is a validated survey [27,28,29] that asks participants to report their level of liking or disliking a variety of foods and beverages from food groups, such as fruits and vegetables, low-fat and high-fat proteins, sweets, fiber, healthy fats, high fat, and refined carbohydrates, as well as activities such as exercise and sedentary behaviors. The responses were scored between −100 to 100 as: “hate it/love it” scoring ±75 to 100, “really dislike/like it” scoring ±45 to 75, “dislike/like it” scoring ±15 to 45, and “it’s ok” scoring −15 to 15 points [31]. The average of responses for each food group was computed and weighted in accordance with the Dietary Guidelines 2020 [30]: vegetables (+3), fruits (+2), fiber foods (+3), high-fat foods (−3), healthy fats (+2), high-fat proteins (−3), low-fat proteins (+3), sweets (−3), and refined carbohydrates (−3).

### 2.4. Assessment of Food Management Skills

Food resource management (FRM) skills were assessed by single-item skills consisting of the validated items on the Expanded Food and Nutrition Education Program (EFNEP) behavior checklist [11]. FRM was assessed based on skills including having a meal plan, importance of reading nutrition labels, comparing prices before buying foods, shopping with a food list, and managing food budget to avoid running out of food (Table 1).

### 2.5. Assessment of Healthy Eating Focus

Healthy eating focus was assessed by self-rated diet quality status [32] and self-reported importance of a healthy diet (Table 1).

### 2.6. Assessment of the Impact of COVID-19 Pandemic on Changes in Health-Related Behaviors

The survey also contained questions about changes in several food behaviors during the pandemic, such as intake of fresh fruits and vegetables, canned or frozen fruits and vegetables, lean meats, chicken, turkey, and pork chops, candy and snack chips, low-fat dairy products, and sugary drinks, including sports drinks and juice drinks. Other questions included changing the frequency of food shopping and eating out, the frequency of exercise, body weight, smoking habits, and household income during the COVID-19 pandemic. Participants were asked to report if they had decreased, not changed, or increased their consumption of these food items and other behaviors. These responses were converted to −1, 0, and 1, respectively, and averaged for the analyses.

### 2.7. Statistical Analysis

Participants were categorized based on tertiles of sHEI and Liking-DQI and the lowest tertiles were considered the reference category. The distribution of sociodemographic and health status variables across tertiles of diet quality measures were calculated using χ2 tests. To examine diet quality measures across categories of FRM skills and healthy eating focus, one-way ANOVA was applied for the crude model and ANCOVA was used to adjust for age, gender, race, physical activity, and overweight/obesity. We assessed the underlying structure of healthy and unhealthy behaviors by exploratory principal components analysis (PCA). Based on the correlations among the 12 variables of health-related behaviors changes with the pandemic, two components were identified: pandemic-related unhealthy behavior (consumption of candy and snack chips, soda and sugary drinks, increased body weight, smoking) and pandemic-related healthy behavior (intake of fresh fruits and vegetables, canned fruits and vegetables, lean meat, low-fat dairy). These components approximated a normal distribution upon visual inspection but only fair internal reliability (alpha unhealthy behavior = 0.59, alpha healthy behavior = 0.49). To assess changes in healthy and unhealthy behaviors during the pandemic across categories of the importance of reading food labels and self-rated diet quality status, one-way ANOVA was used in the crude model and ANCOVA was applied in the model adjusted for age, gender, race, and physical activity. Finally, associations between diet quality measures and likelihood of overweight and obesity, hypertension, and diabetes were calculated using logistic regression in the crude and multivariable models. Analyses were performed using SAS (Version 9.4, SAS Institute, Cary, NC, USA), except for PCA performed using SPSS software (version 28.0, SPSS Inc., Chicago, IL, USA). *p*-values were considered statistically significant at <0.05.

## 3. Results

### 3.1. Sociodemographic and Health Characteristics

As shown in Table 2, there were some differences in the participants’ characteristics across tertiles of sHEI and Liking-DQI indices. Participants aged 40 years or above were more likely to have a higher Liking-DQI (*p* < 0.001) and there were fewer English speakers in the highest tertile of Liking-DQI (*p* < 0.05). The number of women was higher (*p* < 0.001) and participants who were current smokers, and overweight/obese, were lower in the highest tertile of sHEI (*p* < 0.05). There were more physically active participants (*p* < 0.05) in the highest tertile of both diet quality indices compared to the lowest tertile.

### 3.2. The Association of FRM and Healthy Eating Focus with Diet Quality

Adults who reported greater FRM skills and healthy eating focus generally reported healthier diet qualities as indicated by higher sHEI and Liking-DQI scores in both crude and adjusted models (Table 3). After adjusting for age, gender, race, physical activity, and overweight/obesity, participants who usually or always had a meal plan (*p* < 0.001 for sHEI and *p* < 0.01 for Liking-DQI) indicated greater importance of reading nutrition labels (*p* < 0.001), usually or always used grocery lists (*p* < 0.01), and had higher diet quality scores. In terms of healthy eating focus, people who were very concerned about choosing healthy foods (*p* < 0.001) and evaluated their diet as very good or excellent (*p* < 0.001) had better diet quality scores.

### 3.3. Association of FRM and Healthy Eating Focus with Self-Reported Changes in Health-Related Behaviors

**Figure 1:**
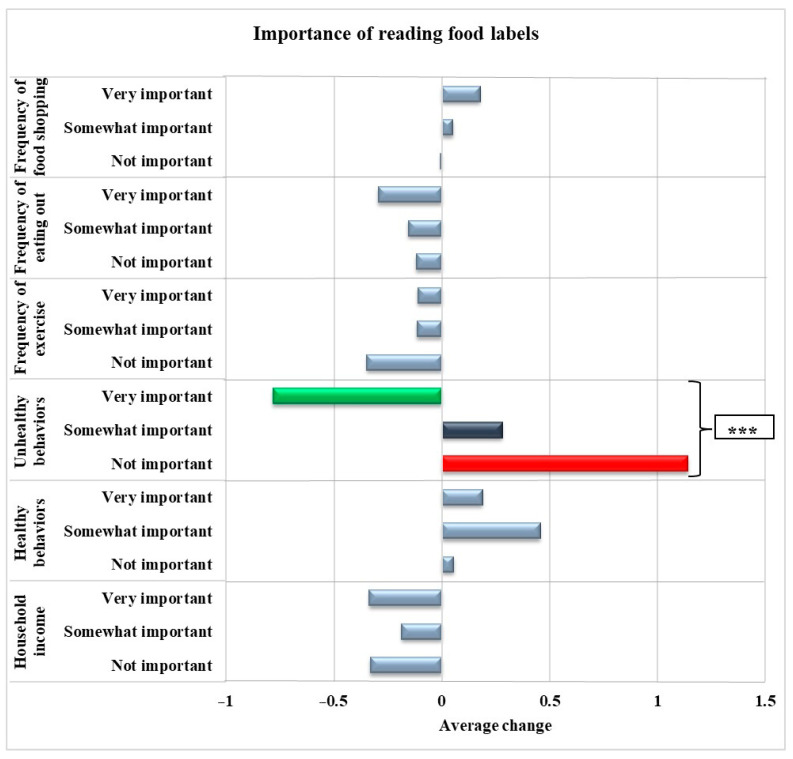
Associations between the importance of reading food labels and pandemic-related healthy and unhealthy behaviors, frequency of shopping, eating out, exercise, and income among adults living in a low-income, low-food-access community in Northeast Connecticut (*n* = 276) adjusted for age, gender, race, and physical activity. *** indicates *p* < 0.001. Unhealthy behaviors: consumption of candy and snack chips, soda and sugary drinks, increased body weight, and smoking. Healthy behaviors: Consumption of fruits and vegetables, canned fruits and vegetables, lean meat, and low-fat dairy.

**Figure 2:**
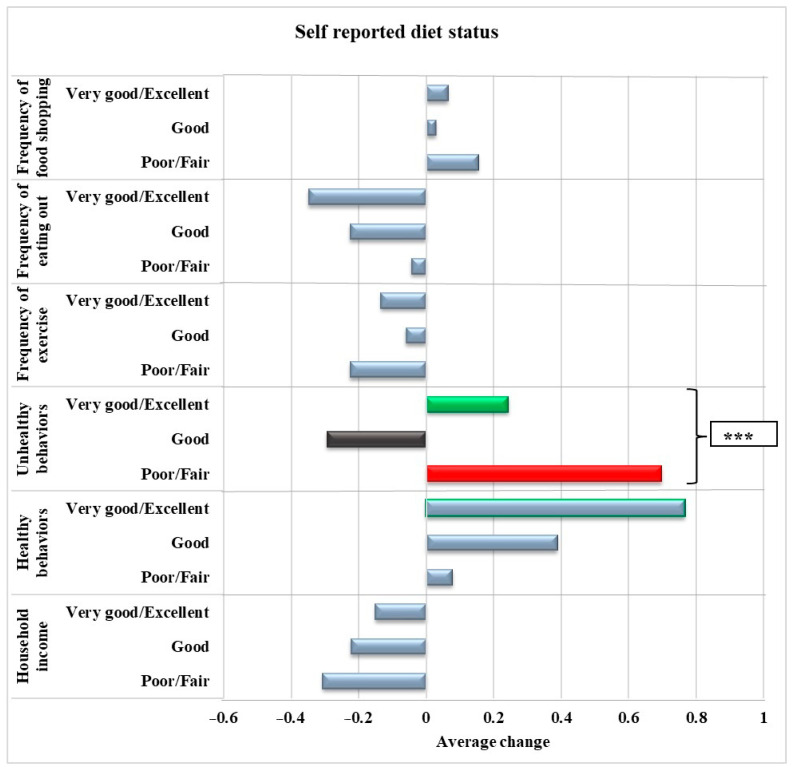
Association between self-reported diet status and pandemic-related healthy and unhealthy behaviors, frequency of shopping, eating out, exercise, and income among adults living in a low-income, low-food-access community in Northeast Connecticut during the pandemic (*n* = 276) adjusted for age, gender, race, and physical activity. *** indicates *p* < 0.001. Unhealthy behaviors: consumption of candy and snack chips, soda and sugary drinks, increased body weight, and smoking. Healthy behaviors: Consumption of fruits and vegetables, canned fruits and vegetables, lean meat, and low-fat dairy.

Figure 1 illustrates the associations between the awareness of the importance of reading food labels and changes in health-related behaviors during the COVID-19 pandemic. After adjusting for age, gender, race, and physical activity, individuals who considered reading food labels very important had a decreased frequency of pandemic-related unhealthy behaviors (*p* < 0.001). Figure 2 displays the association between self-reported diet quality and changes in health-related behaviors during the pandemic in the adjusted model. Adults who reported having a good to excellent diet reported a lower frequency of pandemic-related unhealthy behaviors (*p* < 0.001).

### 3.4. Diet Quality and Risk of Overweight and Obesity, Hypertension, and Diabetes

Table 4 shows associations between the diet quality measures and odds of reporting overweight and obesity, diabetes, and hypertension. The findings showed that there was a significant inverse relationship between higher sHEI scores and the odds of overweight and obesity in both the crude (OR: 0.37; 95% CI: 0.19, 0.74; *p*-trend < 0.01) and adjusted models (OR: 0.37; 95% CI: 0.18, 0.76; *p*-trend < 0.01). No significant associations were found between sHEI and odds of hypertension or diabetes or between Liking-DQI and odds ratios of overweight and obesity, hypertension, or diabetes.

## 4. Discussion

This study highlights relationships between FRM skills, healthy eating focus, dietary quality, and changes in healthy and unhealthy habits during the COVID-19 pandemic among adults from a low-income and low-food-access community in Connecticut. Adults who reported greater FRM skills and had a greater focus on healthy eating reported better diet quality scores. Furthermore, reduction in unhealthy behaviors was reported during the pandemic in adults who were more likely to read food labels and who reported greater diet quality. Finally, adults who reported greater diet quality, by a frequency-based measure (sHEI), had lower odds of being overweight or obese.

The current study found that participants who usually or always planned meals, considered reading nutrition labels very important, and usually or always used shopping lists had better diet quality scores, even after controlling for factors such as age, gender, race, physical activity, and overweight/obesity. These findings are in line with evidence that shows that low-income individuals who are aware of FRM skills have better diet quality [33,34]. Meal planning has been shown to be an efficient strategy to reduce time demands and increase home cooking, which have been associated with better diet quality and lower risk of obesity [35]. Additionally, some studies have demonstrated that reading nutrition labels and having shopping lists are associated with higher diet quality scores, intake of fruits, vegetables, potassium, and fiber, and reduced caloric intake from total fat, saturated fat, cholesterol, and sodium [34,36].

Consistent with the previous literature, the present study confirmed relationships between better healthy eating focus with higher diet quality scores. Sullivan et al. conducted a cross-sectional study using NHANES data and found a positive correlation between self-rated diet quality and total Healthy Eating Index-2015 score and its components, with the exception of dairy and sodium [10]. Another study showed that having a positive attitude regarding healthy eating is connected to higher dietary quality among individuals who shop at supermarkets [37].

Notably, the associations between FRM skills and healthy eating focus with diet quality were seen, whether generating a diet quality score from a frequency survey (sHEI) or a liking survey (Liking-DQI). However, only the frequency-based diet quality score was inversely associated with self-reported overweight or obesity. Food frequency questionnaires are prone to inaccuracy, have measurement bias, and data entry is a time-consuming process for researchers and is subject to data entry error [38]. Short HEI and Liking-DQI surveys are short tools for diet quality assessment which are practical and simple approaches for community-based research. The sHEI tool can accurately estimate some individual nutrient and food group intakes (vegetables, fruits, dairy, sugar-sweetened beverages, added sugar, and calcium) as well as diet quality [26]. The 22-item tool is less burdensome for both the respondents and researchers compared to other common dietary quality assessment methods [26]. The Liking-DQI correlates with indicators of chronic diseases, has favorable psychometric properties, and improves accuracy in dietary assessment and relationships with the likelihood of chronic diseases [27,28,29,39].

A systematic review showed that the COVID-19 pandemic led to a shift towards increasing unhealthy eating behaviors, including an increased frequency of snack and alcohol consumption, greater preference for sweets and ultra-processed food, and decreased preference for fruits, vegetables, and fresh food [13]. Another review indicated that decreased physical activity and sleep quality, as well as increased snacking frequency and alcohol consumption, during the pandemic led to weight gain [40]. These changes emphasize the need for nutrition education to improve pandemic-related unhealthy behaviors. The current study found that participants who considered reading food labels very important had a decreased frequency in unhealthy food consumption, such as candy and snack chips, soda or sugary drinks, smoking, and the least increase in body weight during the pandemic. Furthermore, adults who reported having a good to excellent diet had less of an increase in candy and snack chips consumption and body weight and a decrease in soda and sugary drinks consumption. In accordance with these findings, previous studies showed that reading nutrition labels as well as higher self-rated diet quality are associated with a decrease in the frequency of sugar intake, fast-food dining, and likelihood of obesity [10,41,42,43,44]. These findings highlight the importance of FRM and healthy eating focus in improving eating behaviors, maintaining body weight, and reducing the frequency of smoking during the pandemic. This is particularly important now, given rising food prices and reduction in access.

This is the first study to assess the association of FRM and healthy eating focus with diet quality and changes in health-related behaviors in adults who live in a low-income, low-food-access community in Northeast Connecticut during the pandemic. Participants reported high participation in food assistance programs in this study. Previous studies showed that food assistance program participation decreases food insecurity, without improvements in diet quality [3,4,5,6]. This study showed the need for FRM and awareness of healthy eating as main components of nutrition education programs in addition to federal assistance programs to improve diet quality and reduce unhealthy behaviors. Thus, integrating nutrition education programs and FRM and healthy eating focus into existing food assistance programs can enhance their effectiveness by improving diet quality, which in turn improves nutrition literacy and reduces diet-related chronic diseases. This study has some limitations. First, the convenience sample may have under- or over-represented population groups; however, the demographic characteristics of our sample closely align with those specific zip codes [17,18]. Second, self-reported data were used, which may be prone to over- and under-reporting. Third, due to the cross-sectional nature of this study, causality cannot be detected, and measured change in pandemic-related behavior could not be assessed. While FRM may improve diet, it also may be that people who are the most concerned about their diet are more likely to implement FRM skills. Fourth, 52.2 percent of the study population are White or Caucasian and 74.6 percent are women. This is likely due to the use of an online survey, which may have introduced selection bias towards individuals with internet access, digital devices, and technological literacy. Fifth, composite scores of healthy and unhealthy behaviors did not show good internal reliability and variability (alpha unhealthy behavior = 0.59, alpha healthy behavior = 0.49). This may be due to multidimensionality and complexity of healthy and unhealthy behaviors, and measurement errors. However, in line with our findings, one study also mentioned that internal consistency is not a mandatory characteristic of the HEI [45]. Sixth, although factors previously linked to diet quality were adjusted, unmeasured confounding variables, including energy intake, could affect the findings. The major limitation of frequency-based healthy eating indexes like sHEI is that higher scores are associated with higher energy intake; that is, a higher sHEI could really reflect eating more, higher weight, and hypertension. Thus, one needs to be careful when making any conclusions about the HEI and health measures. Finally, the participants indicated whether they read food labels, but measurement of the level of understanding and/or how their food purchasing habits were adjusted after reading labels was not assessed in this study and should be further evaluated in the future.

## 5. Conclusions

This study showed that food resource management (FRM) skills and healthy eating focus are associated with better diet quality among low-income individuals, reiterating the importance of nutrition education efforts for low-income populations in FRM to support nutrition security. During the COVID-19 pandemic, integrated FRM skills and healthy eating focus, as indicated by importance of reading nutrition label and self-rated diet quality, might be effective to improve eating behaviors, maintain body weight, and decrease smoking among low-income adults. Moreover, higher-frequency-based diet quality index (sHEI) was associated with lower chances of overweight or obesity in these low-income individuals. Considering the benefits of FRM skills and healthy eating focus for improved diet quality, moving forward, these skills should be considered essential components to include in nutrition education targeting low-income populations.

## Figures and Tables

**Table 1:** Single-item food resource management (FRM) skills and healthy eating focus among adults (*n* = 276) living in a low-income, low-food-access community in Northeast Connecticut.

	Survey Item	Item Range	Scores of Study Sample
FRM skills			Mean ± SD
Plan meals	How often does your family eat evening meals together?	1–6 ^1^	4.57 ± 1.64
Importance of reading nutrition labels	When you shop for food, how important is the nutrition label to you? For example, the amount of sodium or sugars.	1–3 ^2^	2.16 ± 0.65
Compare prices	When you shop for food, how important is cost to you?	1–3 ^2^	2.66 ± 0.53
Grocery list	When you shop for groceries, how often do you use a list?	1–4 ^3^	2.81 ± 1.03
Run out of food	Within the past month, did the food you bought last and did you have money to buy more?	1–3 ^4^	2.36 ± 0.77
Healthy eating focus			
Healthy food	How concerned are you about how healthy your food is?	1–3 ^5^	2.07 ± 0.71
Self-evaluated diet	How would you rate your overall diet quality?	1–3 ^6^	1.66 ± 0.70

^1^ Response options: 1 = never, 2 = occasionally/monthly, 3 = couple of times per month, 4 = weekly, 5 = couple of times per week, 6 = usually/always. ^2^ Response options: 1 = not important, 2 = somewhat important, 3 = very important. ^3^ Response options: 1 = never, 2 = occasionally, 3 = sometimes, 4 = usually/always. ^4^ Response options: 0 = I do not know, 1 = never, 2 = sometimes, 3 = often. ^5^ Response options: 1 = not at all concerned/not too concerned, 2 = somewhat concerned, 3 = very concerned. ^6^ Response options: 1 = poor/fair, 2 = good, 3 = very good/excellent.

**Table 2:** Sociodemographic and health characteristics of 276 adults living in a low-income, low-food-access community in Northeast Connecticut by tertile of short Healthy Eating Index (sHEI) and Liking diet quality index (Liking-DQI) ^1^.

	sHEI Tertile ^1^			*p*-Value ^2^	Liking-DQI Tertile ^1^			*p*-Value ^2^
	T1 (*n* = 87)	T2 (*n* = 88)	T3 (*n* = 88)		T1 (*n* = 90)	T2 (*n* = 90)	T3 (*n* = 90)	
	*n* (%)	*n* (%)	*n* (%)		*n* (%)	*n* (%)	*n* (%)	
Gender				<0.001				0.054
Male	29 (11)	30 (11.4)	7 (2.7)		22 (8.2)	30 (11.1)	16 (5.9)	
Female	58 (22)	58 (22.1)	81 (30.8)		68 (25.2)	60 (22.2)	74 (27.4)	
Age				0.28				<0.001
19–39	61 (23.2)	70 (26.6)	58 (22.1)		76 (28.2)	66 (24.4)	52 (19.3)	
40–49	21 (8.0)	15 (5.7)	22 (8.4)		14 (5.2)	18 (6.7)	29 (10.7)	
60+	5 (1.9)	3 (1.1)	8 (3.0)		0 (0)	6 (2.2)	9 (3.3)	
Race/ ethnicity				0.59				0.69
White/Caucasian	48 (18.3)	45 (17.1)	46 (17.5)		48 (17.8)	49 (18.1)	45 (16.7)	
Black/African American	3 (1.1)	9 (3.4)	4 (1.5)		2 (0.7)	7 (2.6)	7 (2.6)	
Latino/Hispanic	31 (11.8)	31 (11.8)	33 (12.6)		35 (13.0)	30 (11.1)	34 (12.6)	
Others ^3^	5 (1.9)	3 (1.1)	5 (1.9)		5 (1.8)	4 (1.5)	4 (1.5)	
Primary Language				0.76				<0.05
English	78 (29.7)	77 (29.3)	80 (30.4)		87 (32.2)	79 (29.3)	77 (28.5)	
Non-English	9 (3.4)	11 (4.2)	7 (3)		3 (1.1)	11 (4.1)	13 (4.8)	
Education				0.06				0.22
≤8th grade/Some High School	8 (3.1)	15 (5.7)	5 (1.9)		8 (3.0)	13 (4.8)	9 (3.3)	
H.S. graduate/ GED	28 (10.6)	22 (8.4)	20 (7.6)		32 (11.8)	21 (7.8)	18 (6.7)	
Some college or technical	30 (11.4)	31 (11.8)	29 (11)		25 (9.3)	33 (12.2)	34 (12.6)	
Graduate/ professional degree	21 (8.0)	20 (7.6)	34 (12.9)		25 (9.3)	23 (8.5)	29 (10.7)	
Employment				0.77				0.35
Full-time employment	44 (16.7)	42 (16.0)	39 (14.8)		42 (15.5)	49 (18.1)	39 (14.4)	
Part-time employment	13 (4.9)	20 (7.6)	18 (6.9)		20 (7.4)	18(6.7)	13 (4.8)	
Unemployed, active seeking	12 (4.6)	10 (3.8)	9 (3.4)		10 (3.7)	8 (3.0)	14 (5.2)	
Unemployed, not seeking	18 (6.8)	16 (6.1)	22 (8.4)		18 (6.7)	15 (5.6)	24 (8.9)	
Cigarette smoking				<0.05				0.18
Current (inc. e-cigarettes)	21 (8)	29 (11)	13 (4.9)		27 (10)	23 (8.5)	16 (5.9)	
Former	10 (3.8)	16 (6.1)	16 (6.1)		11 (4.1)	12 (4.4)	20 (7.4)	
Never	56 (21.3)	43 (16.4)	59 (22.4)		52 (19.3)	55 (20.4)	54 (20)	
Physical activity/week				<0.05				<0.05
<150 min	82 (31.2)	72 (27.4)	71 (27)		79 (29.3)	82 (30.4)	69 (25.5)	
≥150 min	5 (1.9)	16 (6.1)	17 (6.4)		11 (4.1)	8 (2.9)	21 (7.8)	
Overweight and obesity				<0.05				0.76
Yes	69 (26.3)	64 (24.4)	53 (20.2)		60 (22.3)	65 (24.1)	64 (23.8)	
No	17 (6.5)	24 (9.2)	35 (13.4)		29 (10.8)	25 (9.3)	26 (9.7)	
Diabetes				0.28				0.07
Yes	11 (4.2)	8 (3)	5 (1.9)		5 (1.9)	13 (4.8)	6 (2.2)	
No	76 (28.9)	80 (30.4)	83 (31.6)		85 (31.5)	77 (28.5)	84 (31.1)	
Hypertension				0.19				0.22
Yes	23 (8.8)	16 (6.1)	14 (5.3)		16 (5.9)	22 (8.2)	13 (4.8)	
No	64 (24.3)	72 (27.4)	74 (28.1)		74 (27.4)	68 (25.2)	77 (28.5)	

^1^ The means of the short Healthy Eating Index (sHEI) for tertile 1, 2, and 3 are 38.9, 50.2, and 60.2, and the means of the Liking diet quality index (Liking-DQI) for tertile 1, 2, and 3 are −38.16, −1.88, and 32.51, respectively. ^2^ All *p*-values were calculated by χ2 test. ^3^ Includes Asian/South Asian/Pacific Islander, American Indian or Alaskan Native, or multiracial.

**Table 3:** Crude and adjusted measures of diet quality assessed by short Healthy Eating Index (sHEI) and Liking diet quality index (Liking-DQI) by single-item food resource management (FRM) skills and healthy eating focus among adults living in a low-income, low-food-access community in Northeast Connecticut (*n* = 276).

FRM Skills and Healthy Eating Focus	sHEI (*n* = 263)		Liking-DQI (*n* = 270)	
	Crude	Adjusted ^2^	Crude	Adjusted ^2^
	Mean ± SD	Mean ± SE	Mean ± SD	Mean ± SE
Plan meals				
Never	39.27 ± 9.52	40.91 ± 2.49	−21.35 ± 31.65	−16.82 ± 8.09
Occasionally/monthly	51.13 ± 6.34	52.27 ± 2.02	−1.36 ± 34.42	4.94 ± 7.60
Couple of times per month	47.80 ± 8.90	49.08 ± 2.10	−3.95± 33.01	1.65 ± 7.23
Weekly	49.41 ± 7.65	52.16 ± 1.83	−15.62 ± 32.94	−5.11 ± 6.53
Couple of times per week	48.43± 9.83	49.68 ± 1.57	−4.42 ± 34.41	−1.49 ± 5.67
Usually/always	52.11 ± 10.05	53.50 ± 1.27	5.63 ± 32.53	10.80 ± 4.54
*p*-value ^1^	<0.001	<0.001	<0.01	<0.01
Importance of reading nutrition labels				
Not important	41.72 ± 10.27	43.80 ± 1.85	−21.43 ± 34.90	−14.21 ± 6.28
Somewhat important	50.10 ± 8.83	51.46 ± 1.13	−1.62 ± 33.15	2.97 ± 4.08
Very important	52.50 ± 9.52	54.34 ± 1.41	4.88 ± 32.00	11.28 ± 5.01
*p*-value	<0.001	<0.001	<0.001	<0.001
Compare prices				
Not important	51.34 ± 8.65	54.83 ± 4.38	−28.71 ± 22.54	−13.27 ± 13.20
Somewhat important	51.12 ± 9.65	52.54 ± 1.33	−4.70± 36.64	0.50 ± 4.67
Very important	49.19 ± 9.82	50.57 ± 1.28	−0.63 ± 32.85	5.72 ± 4.42
*p*-value	0.33	0.22	0.08	0.20
Grocery List				
Never	46.38 ± 11.00	48.52 ± 1.90	−12.60 ± 40.63	−5.95 ± 6.54
Occasionally	47.50 ± 8.53	49.52 ± 1.38	−9.64 ± 32.57	−1.90 ± 4.91
Sometimes	52.16 ± 9.04	54.33 ± 1.48	−4.40 ± 33.38	5.54 ± 5.09
Usually/always	51.24 ± 10.20	52.91 ± 1.47	8.18 ± 30.57	13.22 ± 5.16
*p*-value	<0.01	<0.01	<0.01	<0.01
Run out of food				
Often	50.11 ± 11.02	51.07 ± 2.06	0.30 ± 31.53	5.80 ± 6.92
Sometimes	49.91 ± 8.90	51.79 ± 1.42	−3.75 ± 31.70	4.00 ± 4.92
Never	49.81± 9.96	51.27 ± 1.24	−2.59 ± 36.24	1.56 ± 4.42
*p*-value	0.99	0.90	0.85	0.76
Healthy Food				
Not at all concerned/not too concerned	46.19 ± 9.73	48.08 ± 1.44	−14.57 ± 30.49	−7.95 ± 4.86
Somewhat concerned	49.31 ± 9.48	51.45 ± 1.32	−6.27 ± 34.93	1.34 ± 4.59
Very concerned	53.17 ± 9.28	55.43 ± 1.42	13.30 ± 29.13	19.63 ± 4.93
*p*-value	<0.001	<0.001	<0.001	<0.001
Self-evaluated diet				
Poor/fair	45.48 ± 9.53	45.71 ± 1.31	−11.81 ± 35.07	−7.84 ± 4.77
Good	53.44 ± 8.37	53.62 ± 1.15	5.54 ± 32.23	7.31 ± 4.32
Very good/Excellent	54.03 ± 8.16	54.21 ± 1.67	6.40 ± 26.46	10.99 ± 6.22
*p*-value	<0.001	<0.001	<0.001	<0.001

^1^ *p*-values were calculated by one-way ANOVA for crude model and ANCOVA for adjusted model. ^2^ Adjusted for age, gender, race, physical activity, overweight, and obesity.

**Table 4:** Odds ratios of overweight and obesity, hypertension, and diabetes by tertile of short Healthy Eating Index (sHEI) and Liking-based diet quality index (Liking-DQI) among adults (*n* = 276) living in a low-income, low-food-access community in Northeast Connecticut ^1,2^.

	sHEI			*p*-Trend
	T1 (*n* = 87)	T2 (*n* = 88)	T3 (*n* = 88)	
Overweight and obesity				
Unadjusted	1.00	0.66 (0.32–1.33)	0.37 (0.19–0.74)	<0.01
Adjusted ^3^	1.00	0.71 (0.34–1.49)	0.37 (0.18–0.76)	<0.01
Hypertension				
Unadjusted	1.00	0.62 (0.30–1.27)	0.53 (0.25–1.11)	0.08
Adjusted	1.00	0.80 (0.36–1.77)	0.61 (0.26–1.39)	0.24
Diabetes				
Unadjusted	1.00	0.69 (0.26–1.81)	0.42 (0.14–1.25)	0.11
Adjusted	1.00	1.05 (0.37–3.03)	0.38 (0.12–1.26)	0.13
Liking-DQI				*p*-trend
	T1 (*n* = 90)	T2 (*n* = 90)	T3 (*n* = 90)	
Overweight and obesity				
Unadjusted	1.00	1.26 (0.66–2.38)	1.19 (0.63–2.25)	0.59
Adjusted	1.00	1.04 (0.54–2.03)	1.12 (0.56–2.24)	0.74
Hypertension				
Unadjusted	1.00	1.50 (0.73–3.08)	0.78 (0.35–1.73)	0.57
Adjusted	1.00	1.16 (0.53–2.54)	0.52 (0.21–1.31)	0.18
Diabetes				
Unadjusted	1.00	2.87 (0.98–8.42)	1.21 (0.36–4.13)	0.79
Adjusted	1.00	2.52 (0.82–7.77)	0.82 (0.22–3.12)	0.74

^1^ The means of the short Healthy Eating Index (sHEI) for tertile 1, 2, and 3 are 38.9, 50.2, and 60.2 and the means of the Liking diet quality index (Liking-DQI) for tertile 1, 2, and 3 are −38.16, −1.88, and 32.51, respectively. ^2^ Odds ratios were calculated by logistic regression. ^3^ Adjusted for age, gender, race, physical activity.

## Data Availability

The data presented in this study are available on request from the corresponding authors.
